# Diagnostic challenges in a patient with dengue shock syndrome presenting with acute meningoencephalitis

**DOI:** 10.1016/j.idcr.2024.e01964

**Published:** 2024-04-15

**Authors:** Kaiho Hirata, Takuyo Chiba, Harumi Gomi, Saho Takaya, Yasuyuki Kato, Takashi Shiga

**Affiliations:** a Department of Emergency Medicine, International University of Health and Welfare Narita Hospital, 852 Hatakeda, Narita City, Chiba 286-8520, Japan; b Center for Infectious Diseases, International University of Health and Welfare Narita Hospital, 852 Hatakeda, Narita City, Chiba 286-8520, Japan; c Office of Medical Education, International University of Health and Welfare School of Medicine, 4-3, Kozunomori, Narita, Chiba 286-8686, Japan

**Keywords:** Dengue virus, Dengue shock syndrome, Dengue meningoencephalitis

## Abstract

Dengue is a systemic viral infection, and clinical findings vary from asymptomatic to life-threatening, including shock and neurological complications. Despite efforts in vector control, the disease continues to spread worldwide, and the number of annual dengue infections is estimated to be 390 million. For patients with severe dengue, early diagnosis is important; however, owing to the wide range of symptoms and severity, diagnosis can be difficult. Herein, we report the case of a 24-year-old man from Vietnam who was found to have dengue shock syndrome complicated by meningoencephalitis, even though he did not show the typical clinical manifestations of dengue infection. He was transported to our hospital by ambulance because of fever and altered mental status. Brain magnetic resonance imaging revealed hyperintensities in the bilateral thalamus and brainstem on the T2 sequence. After hospitalization, polymerase chain reaction testing of cerebrospinal fluid, serum, and urine revealed the presence of dengue virus serotype 2. This confirmed the diagnosis of dengue encephalitis. The patient was discharged on day 49 with impaired abduction of the left eye and urinary retention. In this case, the initial differential diagnosis was broad because the patient was unable to provide any medical history owing to altered mental status. In addition, the fact that he did not show the characteristic symptoms of dengue infection initially made the diagnosis very difficult. In conclusion, dengue fever should always be considered as a part of the differential diagnosis when a patient from an endemic area presents with fever and impaired consciousness.

## Introduction

Dengue fever is a febrile systemic illness caused by the dengue virus, a member of the Flaviviridae family. Dengue virus is endemic in tropical and subtropical regions and is transmitted through the bite of an infected *Aedes Aegypti* mosquito [1]. According to the report form National Institute of Infectious Diseases in Japan, the annual number of cases is less than one hundred since 2020. The number of reported patients with Dengue fever is higher in the months between July and October and is the highest in August.

Typical clinical manifestations include fever, arthralgia, myalgia, and skin rashes. Laboratory findings include hemorrhagic tendencies and liver dysfunction, and most immunocompetent patients recover without complications after symptomatic treatment [2]. However, some patients may develop various severe complications, including neurological complications, and may undergo life-threatening courses. Dengue encephalitis is a neurological complication caused by central nervous system invasion due to the neurotropic effects of the dengue virus. Dengue encephalitis is characterized by altered levels of consciousness, seizures, and focal neurologic dysfunction [3].

We report the case of a patient who was traveling from an endemic area without typical initial clinical manifestations of dengue virus to highlight the importance of raising awareness and suspicion of dengue infection among clinicians.

## Case

**Fig. 1:**
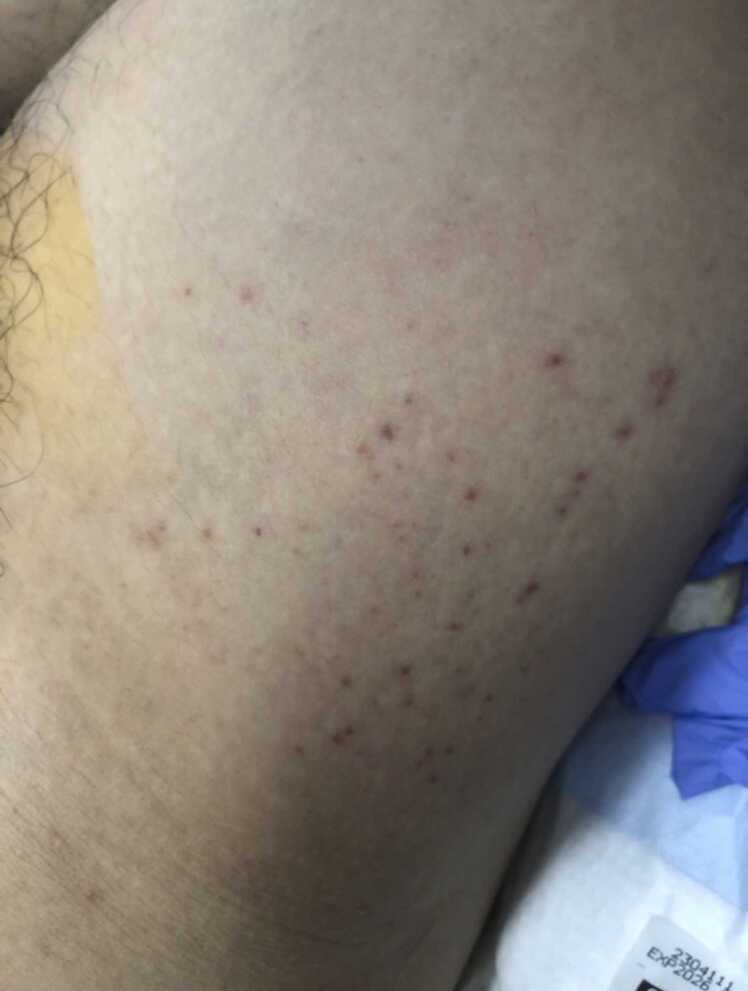
Petechiae on the anterior surface of his right groin and thigh.

A 24-year-old man presented to our emergency department with fever and altered mental status shortly after his flight from Vietnam to an international airport near our hospital on day 1. His initial vital signs were as follows: temperature 41.1 ℃; blood pressure 149/111 mmHg; heart rate 114 bpm; and oxygen saturation were 96 % on room air. Physical examination revealed bilateral conjunctival injection and petechiae on the anterior surface of the right groin and thigh (Fig. 1). His Glasgow Coma Scale score was 9 (E3V2M4), and pupils were equal and reactive to light. At the time, only limited information was available regarding the history of the present illness and the patient’s past medical history because of his altered mental status and the lack of collateral information from his friends or family. Laboratory testing results were significant for a white blood cell count of 8620/μl, C-reactive protein of 5.14 mg/dL, aspartate aminotransferase of 145 U/L and alanine aminotransferase of 137 U/L (Table 1). Lumbar puncture was performed, and dexamethasone and empirical antimicrobial therapy (ceftriaxone, vancomycin, and acyclovir) were initiated. Cerebrospinal fluid showed an elevated protein level (305.9 mg/dL with normal cell counts and glucose levels) (Table 2). Computed tomography (CT) of the head without contrast showed no abnormal findings that would imply the cause of his symptoms. Due to the deteriorating mental status and blood pressure decreasing to 81/59 mmHg, he was intubated and administered intravenous norepinephrine. He experienced a brief episode of seizures for approximately a minute just prior to intubation. Levetiracetam was initiated to treat and prevent the seizures. The patient was admitted to the intensive care unit for further examination and treatment.

**Table 1:** Laboratory data.

Variable	Reference range	Day 0	Day 2	Day 5	Day 10
White-cell count (per μl)	3300–8600	8620	3660	6700	7130
Hemoglobin (g/dl)	13.7–16.8	15.1	12.9	13.9	15.3
Hematocrit (%)	40.7–50.1	44	38.2	39.9	44.7
Platelet count (per μl)	158,000–348,000	156,000	35,000	16,000	237,000
Blood urea nitrogen (mg/dl)	8–20	29	16.1	24.5	20.2
Creatinine (mg/dl)	0.65–1.07	1.73	1.01	0.78	0.81
Glucose (mg/dl)	73–109	110	168	99	94
Albumin (g/dl)	4.1–5.1	4.8	2.9	2.7	4.2
Aspartate aminotransferase (U/l)	13–30	145	1946	192	72
Alanine aminotransferase (U/l)	10–42	137	766	356	135
Alkaline phosphatase (U/l)	38–113	72	52	49	73
Total bilirubin (mg/dl)	0.4–1.5	0.6	0.4	0.7	1
C-reactive protein (mg/l)	0.0–0.14	5.14	1.67	0.33	0.46
Lactate dehydrogenase (U/l)	124–222	441	1543	736	-
Lactate (mmol/l)	0.5–2.2	1.9	3.1	1.5	-
Procalcitonin (ng/ml)	< 0.05	13.2	-	-	-

**Table 2:** Cerebrospinal fluid data.

Variable	Reference range	Day 1	Day 7
Cell count (per μl)	0–5	4	2
Lymphocyte count (per μl)	0–5	3	2
Polymorphonuclear neutrophil count (per μl)	0–5	1	< 1
Glucose (mg/dL)	50–80	75	44
Protein (mg/dL)	10–35	305.9	47.8

On day 3, the liver enzymes AST and ALT were elevated to 1946 and 766 U/L, respectively (Table 1). As the patient was a Hepatitis B carrier, as shown by the blood test on day 1 (positive for hepatitis B surface antigen), hepatitis B virus reactivation due to dexamethasone treatment was the most likely cause of this significant elevation. Soon after dexamethasone was discontinued and entecavir treatment was initiated, the elevated liver enzyme levels improved dramatically.

**Fig. 2:**
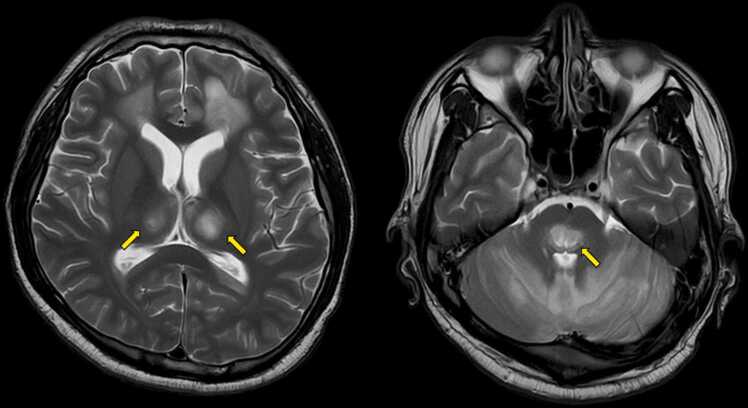
Brain MRI on T2 sequence showing hyperintensities in bilateral thalamus and pons on T2.

On day 7, to submit cerebrospinal fluid samples for polymerase chain reaction (PCR) testing, we repeated a lumbar puncture, which showed an improved protein level of 47.8 mg/dL with a normal cell count and glucose level (Table 2). On day 8, blood, urine, cerebrospinal fluid, stool, and pharyngeal swab specimens were sent to the local Department of Health for PCR testing for representative causative viruses of acute encephalitis (Table 3). Dengue virus serotype 2 was identified in blood, urine, and cerebrospinal fluid specimens. On day 9, Magnetic resonance imaging (MRI) of the brain showed hyperintensities in the bilateral thalamus and brainstem on T2 sequence (Fig. 2). Based on these findings, the patient was diagnosed with dengue shock syndrome complicated by meningoencephalitis.

**Table 3:** Polymerase Chain Reaction (PCR) testing for causative viruses for acute encephalitis.

Herpes simplex virus type 1 and type 2
Varicella zoster virus
Epstein-Barr virus
Cytomegalovirus
Human herpesvirus 6 and 7
Adenovirus
Enterovirus
Mumps virus
Japanese encephalitis virus
West Nile virus
Dengue virus serotype1, 2, 3 and 4
Chikungunya virus
Zika virus

The patient’s condition gradually improved during the hospital stay. The patient was extubated on day 8 as his mental status improved. Over the clinical course, his platelet count declined to 1600/μl on day 6, and he received a 20-unit platelet transfusion. On day 10, the patient was transferred to the general ward and underwent rehabilitation. During rehabilitation, impaired abduction of the left eye and urinary retention were observed as sequelae. The patient was discharged on day 49 and sent to a hospital in Vietnam for further rehabilitation.

## Discussion

Neurological complications of dengue fever include encephalopathies, encephalitis, and Guillain-Barre syndrome [4], but these are very rare complications [5]. Patients with dengue encephalitis tend to have systemic manifestations, including fever and hematological and hepatic involvement [6]. However, our patient presented with a few typical manifestations of dengue infection. Initially, the neurological manifestations presented as clinical symptoms, and thrombocytopenia, which is representative of the findings of dengue infection, appeared later. Firstly, the patient was transported to the emergency department by ambulance from an airport complaining of fever and altered mental status. He was diagnosed with shock, and the initial differential diagnoses were fever, septic shock, and meningoencephalitis. This provided clinicians a broader set of differential diagnoses, including Zika virus infection, Chikungunya virus infection and sepsis associated encephalopathy, and difficulties in prioritizing the diagnosis of dengue fever initially.

There are four serotypes of dengue viruses, called dengue virus serotype 1, 2, 3 and 4. Among the four serotypes, each serotype has a cross-protection against the other serotypes. However, the protection is temporary and disappears over several months after an infection. Some papers suggest the relationship between the serotype 2 and 3, and neurological complications [5], [7], [8], [9], [10]. This case of dengue virus serotype 2 infection with neurological complication supports the suggestion.

Recently, Xu et al. proposed that elevated procalcitonin level could be a reliable marker for dengue encephalitis [11]. It was hypothesized that procalcitonin increases the activities of reactive oxygen species, which contributes to blood-brain barrier disruption and neurological complications of dengue infection. In this case, his procalcitonin level was elevated on day 0 (Table 1), which supports the suggestion. In general, it is known that the immune system degenerates by aging and young people have strong immune systems, including cytokines and procalcitonin. In addition, the ages of all nine cases of dengue infection with neurological complications which Pal et al. reported [7] were less than 50 years of age. Based on these facts, younger ages might be associated with developing neurological complications of dengue infection.

In this case, we made the diagnosis of dengue infection using a PCR test of blood and cerebrospinal fluids. However, there are other ways to identify dengue virus infection: IgM antibody and NS1 antigen. The sensitivity of each diagnostic test depends on the disease phase [7]. During the acute phase, PCR test and NS1 antigen test are suitable while IgM antibody may not be detected within the first week of the infection onset. An IgM antibody test costs much less and is easily accessible even in source-limited areas.

In this case, dexamethasone was administered but was discontinued because of the reactivation of the Hepatitis B virus. Therefore, we could not assess the effects of dexamethasone on this disease. There is still no definitive therapy for dengue fever, although Bandara et al. suggested that corticosteroids may be a treatment option for dengue infections [12]. More cases and data are needed to improve treatment methods for dengue fever.

In conclusion, we presented a relatively rare clinical course of dengue virus infection complicated by meningoencephalitis, in which the patient showed neurological manifestations initially, and the typical systemic findings appeared later. Although neurological complications are very rare in dengue virus infection, we should suspect dengue virus infection as part of the differential diagnosis from the beginning in patients with fever and neurological manifestations for those who are from an endemic area of dengue fever. On the basis of our patient with dengue virus infection with neurological complications, a serotype 2 of dengue virus and younger age may have associations with neurological complication.

## Ethical approval

In this study, ethical approval was not required.

## Funding sources/Funding

This study did not receive any specific grants from funding agencies in the public, commercial, or not-for-profit sectors.

## CRediT authorship contribution statement

**Kaiho Hirata:** Conceptualization, Writing – original draft. **Harumi Gomi:** Conceptualization, Writing – review & editing. **Takuyo Chiba, Saho Takaya:** Writing – review & editing. **Yasuyuki Kato, Takashi Shiga:** Writing – review & editing, Supervision.

## CRediT authorship contribution statement

**Takashi Shiga:** Writing – review & editing, Supervision. **Yasuyuki Kato:** Writing – review & editing, Supervision. **Saho Takaya:** Writing – review & editing. **Harumi Gomi:** Writing – review & editing, Conceptualization. **Takuyo Chiba:** Writing – original draft. **Kaiho Hirata:** Writing – original draft, Conceptualization.

## Declaration of Competing Interest

All authors do not have any conflicts of interests.
